# Supplementary material for: MetaRibo-Seq measures translation in microbiomes
Source: Nat Commun. 2020 Jun 29;11:3268. doi: 10.1038/s41467-020-17081-z (PMC7324362; doi:10.1038/s41467-020-17081-z)
Supplement: Supplementary file 10 — Supplementary Data 7 [file 41467_2020_17081_MOESM10_ESM.zip › File2/Confidence_High_Taxonomy/277512_out.krona.html]

Javascript must be enabled to view this page.

members
magnitude
magnitudeUnassigned
count
unassigned
taxon
rank

277512\_out

17

17
superkingdom
2

17
976
phylum

17
200643
class

17
order
171549

815
family
1

genus
816
1


SRS017247\_contig\_number\_3372
821
species
1

family
2005525
13

375288
genus
13

46503
species

SRS020328\_contig\_number\_10802SRS050941\_contig\_number\_6450SRS063489\_contig\_number\_2947SRS064757\_contig\_number\_16553SRS075341\_contig\_number\_9072SRS097958\_contig\_number\_3671SRS1041039\_contig\_number\_8057SRS104311\_contig\_number\_20913SRS1055069\_contig\_number\_5876SRS143148\_contig\_number\_17739SRS144297\_contig\_number\_11582SRS144714\_contig\_number\_27698SRS147557\_contig\_number\_26906
13

3
species
1897039

SRS014287\_contig\_number\_16349SRS054059\_contig\_number\_4997SRS062427\_contig\_number\_3113
